# Big Five Personality Traits, Personal Projects, and Compulsive Buying: A Causal Approach

**DOI:** 10.3390/ejihpe15020019

**Published:** 2025-02-06

**Authors:** José Manuel Otero-López, María José Santiago, María Cristina Castro

**Affiliations:** Department of Clinical Psychology and Psychobiology, Faculty of Psychology, C/Xosé María Suárez Nuñez, s/n, Campus Vida, 15782 Santiago de Compostela, Spain; mariajose.santiago@usc.es (M.J.S.); mariacristina.castro@usc.es (M.C.C.)

**Keywords:** personality, five factor model, personal projects, compulsive buying, path analysis

## Abstract

The purpose of this paper is to predict compulsive buying based on the integration of explanatory units located at different levels of personality analysis (dispositional vs. motivational variables). More specifically, we propose a causal model that examines the extent to which personal projects (particularly, the domains of meaning and stress) channel the influence of the Big Five personality traits on compulsive buying. The results obtained from a structural equation analysis using a sample of 487 university students generally confirm the suitability of this mediating model. Specifically, while the meaning of projects channels the influence of all traits on compulsive buying, neuroticism and conscientiousness have—in addition to a direct influence on compulsive buying—an indirect influence through the stress of personal projects. The findings obtained not only make it possible to better understand the dynamics between personal variables of different nature and compulsive buying but also allow us to suggest some guidelines for preventive action and intervention on this complex problem.

## 1. Introduction

A substantial agreement has been reached between both clinicians and researchers on the fact that for some people, in specific circumstances and contexts, compulsive buying (CB) may become a problem with grave economic, family-related, and personal consequences, to name but a few. This underlines the urgent need to intensify research efforts to clarify its nature and scope.

CB is characterized by an irresistible urge to buy and a loss of control over buying behavior (McElroy et al., 1994), thus becoming a primary response to negative events or feelings (O’Guinn & Faber, 1989). Recent studies have confirmed both an increase in CB (Adamczyk, 2024; Maraz & Yi, 2022) and an increased risk to develop critical shopping behaviors among the young (Black, 2022; Niedermoser et al., 2021). CB prevalence rates among university students in different countries are revealing: 16.1% in South Korea (Koh et al., 2020), 11% in Poland (Tarka & Kukar-Kinney, 2022), 10.4% in China (He et al., 2018), and 7.4% in Spain (Villardefrancos & Otero-López, 2016). Although sociocultural contexts and methodologies can account for the variability in these percentages, these data underscore the high magnitude of this phenomenon. In light of this scenario, making progress in the efficacious identification of the variables that influence the onset, development, and continuation of CB is crucial.

In this regard, although it has been demonstrated that variables of such different nature such as sociodemographic (Mattos et al., 2016), family (J. Roberts et al., 2019), and cultural variables (Maccarrone-Eaglen & Schofield, 2018), contribute to CB, it is also true that, today, a large volume of studies underpin the significant protagonism of personal determinants in this behavior (Otero-López, 2022). For instance, traits like neuroticism (Aksoy et al., 2023) and narcissism (Emin et al., 2024), temperamental variables like impulsivity (Billieux et al., 2008) and sensation-seeking (Lejoyeux et al., 1997), affective variables like depression and anxiety (Otero-López & Villardefrancos, 2013b), motivational variables like materialism (Dittmar, 2005), life aspirations (J. A. Roberts & Pirog, 2004), and personal projects (Otero-López et al., 2021c), and cognitive variables like self-efficacy (Jiang & Shi, 2016), optimism (Villardefrancos & Otero-López, 2016), and self-esteem (De Pasquale et al., 2022) are some of the directions followed by research on the personal variables involved in CB.

Beyond this large variety of personal variables linked to CB, which have often been studied in isolation, the recent emergence of several frameworks for the study of human personality—like McAdams’ tripartite framework (McAdams, 2013; McAdams & Pals, 2006) and The Five Factor Theory (FFT) by McCrae and Costa (2008)—has resulted in new and suggestive avenues for research. These proposals, with their markedly integrative vocation, have underscored the suitability of building bridges between personal units of different nature (e.g., dispositional or motivational). This represents a breakthrough in gaining a more comprehensive understanding of the individual (in this case, the compulsive buyer).

The Five Factor Theory (FFT) (McCrae & Costa, 2008) assumes the existence of personality traits or “basic tendencies” (in a 2013 proposal, McAdams places these units in layer 1—the person as a social actor) which characterize persons through relatively enduring patterns of thoughts, emotions, and actions. Specifically, the FFT posits that traits are said to be organized hierarchically into five broad factors: neuroticism (the tendency to experience negative emotions such as anxiety and depression), extraversion (the tendency to be sociable, warm, active, assertive, cheerful, and seek stimulation), openness to experience (the tendency to be imaginative, creative, unconventional, emotionally and artistically sensitive), agreeableness (the tendency toward altruism, trust, modesty, and cooperativeness), and conscientiousness (the tendency to be organized, strong-willed, persistent, reliable, and a follower of rules and ethical principles). Beyond dispositional traits, FFT refers to characteristic adaptations, which include a broad spectrum of motivational and social–cognitive personal units (e.g., values, goals, strivings, skills, plans). In McAdams’s approach (McAdams, 2013), this type of units makes up layer 2 (the person as a motivated agent), where goals and future-oriented personal action units (e.g., personal strivings, personal projects, current concerns, life tasks) play a prominent role and, unlike traits, are contextualized in terms of time, place, or role. In sum, both proposals place dispositional traits (basic tendencies or units of level I) and motivational or goal-oriented units (characteristic adaptations or level II) in distinct spaces and under different rubrics, suggesting the adoption of integrative perspectives that encompass both types of personality variables. Of particular relevance to this research is that, within the framework of FFT, characteristic adaptations are concrete manifestations of the Big Five basic tendencies.

Among the different personality units under the label of characteristic adaptations, personal projects (PPs) have shown a high heuristic value and have been consolidated as a key personal factor in predicting and explaining human well-being (Little et al., 2007). PPs are defined as “a set of interrelated acts extending over time, which is intended to maintain or attain a state of affairs foreseen by the individual” (Little, 1983, p. 276) and reflect present and future goals that provide people’s lives with sense and coherence. In addition, it is important to note that PPs are a central theoretical element of the Social Ecological Model (SEM) of well-being (Little, 2000). This model posits that “well-being is influenced by stable and dynamic features of both persons and their contexts and that each of these sources of influence is integrated in the personal projects individuals pursue” (Little & Coulombe, 2015, p. 757). PPs, in conjunction with other elements such as human flourishing (e.g., health, accomplishments, and well-being), stable personal features (e.g., traits, abilities, enduring preferences, and orientations), stable contextual features (e.g., cultural norms and economic cycles), dynamic person features (e.g., free traits), and dynamic context features (e.g., personal contexts), make up this framework. Moreover, the SEM puts forward a variety of ways in which its components influence well-being (Little & Balsari-Palsule, 2021); In this regard, it should be noted that although stable person features (traits) and PPs have a direct effect on well-being, an indirect path of influence also emerges for PPs. Among the dimensions of PPs, meaning and stress are the ones which, based on empirical evidence, have shown the strongest link to behavioral problems such as alcohol use (Lecci et al., 2002; Palfai & Ralston, 2011) and marijuana use (Wright & Palfai, 2012).

In short, the emergence in the last few decades of schemas and/or models such as those by McAdams (2013), McCrae and Costa (2008), and Little (2000), which posit the fruitful coexistence of different types of personality units (dispositional vs. motivational units, for example) and suggest relationships between the more stable elements of personality and the more changing ones, is the base for this study. Specifically, and given the lack of causal approaches that link personality units placed at different levels or in different domains within the field of CB, this study puts forward a model of relationships where goal orientation (particularly the dimensions of meaning and stress of PPs) has a mediating role between dispositional elements (specifically the Big Five personality traits) and CB.

A review of the literature on the role of the Big Five in CB confirms that neuroticism and conscientiousness emerge as the traits more closely linked to this behavioral problem. There is extensive empirical evidence that neuroticism is positively and significantly associated with CB (Aksoy et al., 2023; Andreassen et al., 2013; Mowen & Spears, 1999; Otero-López et al., 2017; Rachubińska et al., 2024; Rizzo et al., 2023; Tarka et al., 2022; Tarka & Harnish, 2023). In contrast, conscientiousness has negative correlations with this behavior (Andreassen et al., 2013; Aquino & Lins, 2023; Mowen & Spears, 1999; Otero-López et al., 2017; Rizzo et al., 2023; Tarka et al., 2022; Uzarska et al., 2023). In addition, it has been demonstrated (Otero-López & Villardefrancos Pol, 2013) that all the facets of neuroticism and conscientiousness establish significant differences between groups with different levels of vulnerability to CB (the most salient are anxiety and impulsivity and sense of duty and self-discipline, respectively). As to the three remaining factors—extraversion, openness to experience, and agreeableness—the results obtained in the previous literature are not conclusive. By way of example, high levels of extraversion and openness to experience have been related to increased vulnerability to CB in several studies (Tarka et al., 2022; Tarka & Harnish, 2023), while other studies (Andreassen et al., 2015) confirm that CB has a positive association with extraversion and a negative association with openness to experience. In a study that analyzed both general traits and facets (Otero-López & Villardefrancos Pol, 2013), it was confirmed that, although there were no differences between groups with different vulnerability to CB with regard to extraversion and openness to experience, the facets of emotion seeking, positive emotions, and assertiveness (extraversion) and the facets of aesthetics and ideas (openness to experience) did establish significant differences. As far as agreeableness is concerned, some studies report a negative correlation with CB (Andreassen et al., 2013; Uzarska et al., 2023). Other studies, however, show that this link is positive (Cheema et al., 2014; Mowen & Spears, 1999).

Lastly, as far as the link between PPs and CB is concerned, it is worth noting that there is previous empirical evidence that provides this approach with meaning and coherence. It has been found that the way people assess their PPs is not just linked to their psychological well-being (Little et al., 2007) but also to a variety of problems (e.g., stress, depression, substance use). Thus, people with hypochondriasis perceive themselves as engaging in stressful and difficult health pursuits (Karoly & Lecci, 1993); those diagnosed with anxiety and depression are characterized by a negative assessment pattern characterized by PPs that are high in distress and perceived difficulty (Lecci et al., 1994). Similarly, those who report health complaints consider their PPs as highly stressing (Wallenius, 2007). From the domain of substance addiction, Lecci et al. (2002) show that the distress associated with the pursuit of goals is a risk factor for alcohol-related problems, while the meaning of personal goals is a protective factor. Other studies (Palfai et al., 2011; Palfai & Ralston, 2011; Palfai & Weafer, 2006) have found that lower levels of goal meaning were associated with more alcohol use and alcohol-related negative consequences. As to marijuana use, Wright and Palfai (2012) conclude that students who reported high levels of meaning in their PPs were less likely to use marijuana. Despite the major prominence that PPs have achieved in the study of well-being and psychological distress, as well as in the fields of addiction and drug use, in the domain of CB, there is only one study in the previous literature (Otero-López et al., 2021c) that explores the link of this behavioral problem with PPs. This underscores the suitability of continuing down this avenue of enquiry. The results obtained confirm that it is young people with high vulnerability to CB, as opposed to those with moderate or low levels, who appraise their PPs as more stressful and less significant. More specifically, when compared with the other two groups, high-risk university students were characterized by attaching less importance to their PPs, a lower level of enjoyment and commitment, as well as by appraising their PPs as more challenging, difficult, and as generators of stress.

In essence, a review of the current state of the literature confirms that research shows the existence of links between the Big Five personality traits and CB (Tarka et al., 2022), between the Big Five and PPs (Little et al., 1992), and between PPs and CB (Otero-López et al., 2021c). However, there is no study, to our knowledge, on the joint influence of the Big Five and PPs on explaining CB. To fill this gap, and in an attempt to further our understanding of the dynamics of connections underlying CB, the objective of this study is to empirically confirm a model of causal relations that encompasses the network of connections existing between the Big Five personality traits, the meaning and stress of PPs, and CB. Specifically, our model proposal is supported by the hypothesis that the stress and meaning of PPs have a mediating role in the influence of the Big Five on CB.

## 2. Materials and Methods

### 2.1. Procedure and Participants

This study, conducted in the Autonomous Community of Galicia (Spain), falls within a line of work developed over the last few years aimed at clarifying the nature and scope of the different clusters of personal, social, and contextual variables affecting CB. More specifically, in this study, personality variables such as the Big Five and PPs (the dimensions of meaning and stress) were selected to explain CB. The sample was collected in several schools of the University of Santiago de Compostela. Previously trained members of the research team administered the different self-reports in classrooms. Students were previously informed in detail of the objectives of this study, and emphasis was made on the fact that participation was voluntary. Those students who decided to participate were given assurance of the anonymity and confidentiality of the data (for more details about the procedure, see Otero-López et al., 2021a). This research was carried out in accordance with the Declaration of Helsinki, and the protocol was authorized by the Bioethics Committee of the University of Santiago de Compostela.

The study participants were 487 university students pursuing courses in different schools at public University of Santiago de Compostela in Galicia, Spain. Respondents’ characteristics were as follows: 214 were male (43.9%) and 273 were female (56.1%); their ages ranged between 18 and 23 years (mean age = 18.92 years and SD = 1.02).

### 2.2. Measurements

#### 2.2.1. Compulsive Buying

CB was assessed using the Spanish translated version (Reisch, 2001) of the German Addictive Buying Scale (GABS) (Scherhorn et al., 1990). The GABS is a self-report that includes 16 statements which measure various aspects of CB; examples of items on this scale are “Sometimes I buy something that I cannot afford”, “When I have money, I have to spend it”, “I often feel a sudden, inexplicable urge to go out immediately and buy things that I want”. The items were answered on a four-point Likert scale (1: strongly disagree to 4: strongly agree) and the tendency to engage in CB is given by the sum of all item scores (range: 16–64). GABS has showed good psychometric properties in previous research with samples of young Spanish respondents (Otero-López et al., 2021a, 2023). In this study, Cronbach’s alpha was 0.82.

#### 2.2.2. Big Five Personality Traits

The participants completed the Revised NEO-Personality Inventory (NEO-PI-R) in its Spanish version (Costa & McCrae, 2008) to assess the Big Five personality traits. The NEO-PI-R consists of 240 items answered on a five-point Likert scale format ranging from 0 (strongly disagree) to 4 (strongly agree) that rates the Big Five domains of personality, namely, neuroticism, extraversion, openness to experience, agreeableness, and conscientiousness. The NEO-PI-R has been used in the field of CB with Spanish samples in both the general population (Otero-López & Villardefrancos, 2013a; Otero-López & Villardefrancos Pol, 2013) and university students (Otero-López et al., 2023). Internal consistency indices (Cronbach’s alpha) for the five traits in the current study ranged between 0.82 for openness to experience and 0.90 for neuroticism.

#### 2.2.3. Personal Projects

To assess PPs, a standard format of the Personal Projects Analysis (PPA) (Little, 1983, 1989) translated into Spanish by Otero-López et al. (2013) was used. Participants were required to generate their nine planned or ongoing PPs. Subsequently, in a matrix, each of their elicited PPs were assessed (using a Likert-type scale from 0 to 10) in 17 dimensions (e.g., importance, enjoyment, difficulty, challenge, …), which are grouped into five core domains (meaning, structure, efficacy, communality, and stress). In this study, we only selected the dimensions of meaning and stress of PPs, fundamentally because they have shown greater predictive capacity for other behavioral problems. Specifically, meaning in PPs (whether one’s pursuits are seen as worthwhile or worthless) is defined by the dimensions of importance, enjoyment, value congruency, self-identity, and absorption. The dimensions that include the stress of PPs (whether the demands of our projects exceed our capacity to cope with them) are difficulty, stress, and challenge. The scores obtained for the meaning and stress of PPs were calculated by averaging the participants’ scores across all PPs. As to the internal consistency of the PPA, in line with previous studies (Little & Coulombe, 2015; Otero-López et al., 2021c), moderate Cronbach’s alpha coefficients were obtained for the meaning (0.73) and stress (0.76) of PPs.

### 2.3. Statistical Analysis

Statistical analyses were carried out using the IBM SPSS statistical package version 29. Before conducting the statistical analyses, the assumption of normality for all variables included in this study was satisfied (values of skewness and kurtosis within the ranges suggested in the literature; skewness < 2, kurtosis < 10; Kline, 2015). Complementary visual inspection (e.g., Q-Q plots) confirmed the data’s approximate normality. Subsequently, regression analyses corresponding to the procedure by Baron and Kenny (1986) for mediation effect detection (least squares method) were conducted, with the variables entered simultaneously. On the basis of the corresponding guidelines, the Big Five traits of personality can be assumed to have an effect mediated by the stress and the meaning of PPs, provided that (a) the Big Five personality traits predict the meaning and stress of PPs; (b) the Big Five personality traits predict CB; and (c) when the Big Five personality traits and the meaning and stress of PPs are included in the same regression, the appraisal domains of PPs predict CB, and the effect of Big Five personality traits is lessened or even disappears. Then, a model including significant effects on regression results was tested with path analysis using the AMOS version 29.0 software, and maximum likelihood was used as estimation procedure. The goodness-of-fit of the model was estimated using the chi squared test (χ^2^; *p* > 0.05 indicates acceptable fit), the χ^2^/df ratio, the Comparative Fit Index (CFI; values ≥ 0.90), the Goodness-of-Fit Index (GFI; values ≥ 0.95), the Adjusted Goodness-of-Fit Index (AGFI; values ≥ 0.95), the Normed Fit Index (NFI; values ≥ 0.95), and the Root Mean Square Error of Approximation (RMSEA; values ≤ 0.08) (Hu & Bentler, 1999; Kline, 2015). Correlation coefficients were interpreted according to Cohen (1988): r ≤ 0.30 indicates a weak correlation, r = 0.30–0.50 a moderate correlation, and r ≥ 0.50 a strong correlation. Statistical significance was set at *p* < 0.05.

## 3. Results

The objective of this study was to ascertain whether PPs (particularly the dimensions of meaning and stress) channel the influence of the Big Five personality traits on CB. The results of the first regression analysis (Table 1), corresponding to the procedure by Baron and Kenny (1986)—in which the Big Five personality traits were the independent variables and the meaning and stress of PPs were the criterion variables—confirm that all Big Five personality traits are valid predictors of the meaning of PPs (17.4% of variance explained); moreover, neuroticism, openness to experience, and conscientiousness predict the stress of PPs (12.3% of variance explained).

The second regression equation showed that predictors of CB at statistically significant levels were neuroticism and conscientiousness (26.1% of variance explained). The last regression analysis results, when the Big Five personality traits were jointly included with the meaning and stress of PPs as predictors of CB (35.2% of variance explained), generally supported the mediational hypothesis. Thus, both the meaning and stress of PPs emerged as significant predictors of CB (β = −0.22 and 0.27, respectively). Specifically, the value of extraversion, agreeableness, and openness to experience markedly decreased to the point of not being statistically significant. Overall, the results suggest that the meaning and stress of PPs may somehow capture the effects of the Big Five personality traits on CB, in spite of the fact that, as shown in the third regression analysis, neuroticism and conscientiousness exert a direct influence on CB.

Lastly, based on the results obtained in previous regression analyses and in order to obtain a comprehensive representation of the link between the Big Five personality traits, the meaning and stress of PPs, and CB, we put forward a model that was subjected to empirical testing from path analysis as implemented in AMOS v. 29.0 software. Table 2 includes the correlations, mean values, and standard deviations for the variables included in the analysis.

As shown in Table 2, the strongest correlations (higher than 0.40) correspond to neuroticism and the stress of PPs with CB (r = 0.42 and r = 0.40, respectively, *p* < 0.001). Moreover, moderate correlations can be seen between conscientiousness and CB (r = −0.38, *p* < 0.001) and between the meaning of PPs and CB (r = −0.34, *p* < 0.001).

On the other hand, the results show significant associations between the traits of the Big Five and the meaning of PPs: there is a negative association with neuroticism (r = −0.20, *p* < 0.001) and positive associations with conscientiousness (r = 0.32, *p* < 0.001), agreeableness (r = 0.22, *p* < 0.001), openness to experience (r = 0.17, *p* < 0.001), and extraversion (r = 0.16, *p* < 0.001).

As to the link between personality traits and the stress of PPs, particularly noteworthy are the positive and statistically significant correlations for neuroticism (r = 0.26, *p* < 0.001) and the negative correlations for conscientiousness (r = −0.27, *p* < 0.001), agreeableness (r = −0.15, *p* < 0.001), and openness to experience (r = −0.09, *p* < 0.05).

**Figure 1:**
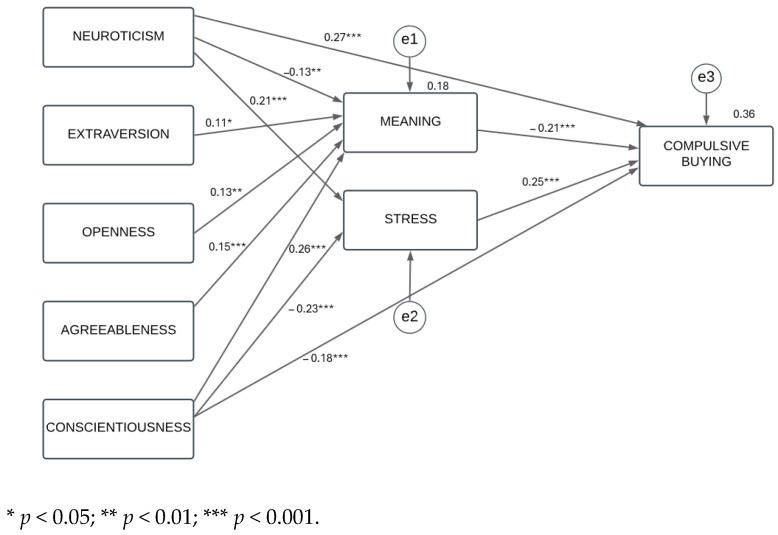
Final model with the relationships between the Big Five, the meaning and stress of PPs, and CB. ***Note*:** e1, e2, and e3 represent the error terms; the values on the arrows are standardized structural estimates with their levels of significance. The values shown above the boxes of the mediating and endogenous variables correspond to the explained variance.

In the model tested, the idea was to map only those relationships that reached statistical significance in the regression analysis results. In this regard, the indirect effects of neuroticism, extraversion, agreeableness, and conscientiousness on CB through the meaning of PPs were emphasized. Similarly, the effects of neuroticism, openness to experience, and conscientiousness on the stress of PPs were represented. The direct influence of neuroticism and conscientiousness on CB was also considered. The results of the path analysis, conducted using the maximum likelihood estimation as the procedure for parameter estimation, are presented in Figure 1.

The goodness-of-fit indices indicate, in general, a good model fit [χ^2^(7) = 13.96, *p* = 0.052; χ^2^/df = 1.99; CFI = 0.99; GFI = 0.99; AGFI = 0.97; NFI = 0.97; RMSEA = 0.04].

In short, the model demonstrated the mediating role of the meaning and stress of PPs in the link between the Big Five personality traits and CB, with direct effects of neuroticism and conscientiousness on CB. Specifically, all Big Five traits exerted indirect effects on CB through the meaning of PPs, whereas the stress of PPs channeled the effect of neuroticism and conscientiousness on CB. Finally, neuroticism and conscientiousness, in addition to the above indirect effects, had a direct effect on this behavioral problem.

## 4. Discussion

Based on the analysis of the previous literature, one of the current challenges of research into CB is the formulation of causal models that include personal units of different nature (including dispositional, purpose-oriented, and goal-oriented units). These units represent different levels or spaces within theoretical schemes with a high heuristic value in the study of personality (Little, 2000; McAdams, 2013; McCrae & Costa, 2008). In view of this need, this study seeks to further the knowledge of CB by empirically analyzing a model of causal relations. In this model, the Big Five (traits or dispositions) are the exogenous variables, personal projects —in particular the dimensions of meaning and stress–act as mediating variables and CB is the endogenous variable.

Generally speaking, the findings obtained confirm the mediating role of the appraisal of PPs in the relation between the Big Five personality traits and CB. The meaning of PPs emerged as the most relevant mediating personal unit as it was found that it channels the influence of the Big Five on this problem. On the other hand, the stress of PPs only channels the link of neuroticism and conscientiousness with CB. In addition, as well as the two indirect paths mentioned above, a direct effect of neuroticism and conscientiousness on CB was confirmed.

### 4.1. The Mediating Role of the Meaning of Personal Projects in the Link Between the Big Five and Compulsive Buying

Meaning, according to our results, has a prominent mediating role between neuroticism and CB. High levels of neuroticism were found to be linked to a low attribution of meaning to PPs, which, in turn, increased the risk of CB. This finding is in line with the pioneering study by Little et al. (1992), who concluded that neuroticism is significantly associated with low scores in some dimensions of the meaning of PPs (identity and enjoyment). As to the link between the meaning of PPs and CB, our results are consistent with those obtained in a recent study (Otero-López et al., 2021c), in which it was found that university students with high vulnerability to CB, compared to those with low involvement, reported lower meaning in their PPs. In other words, they identified less with their PPs, placed less importance on them, reported lower enjoyment in their performance, and showed a lower level of commitment in their accomplishment.

Furthermore, it has been reported in neighboring fields that a low level of meaning of PPs is associated with a higher consumption of alcohol (Lecci et al., 2002; Palfai et al., 2011; Palfai & Ralston, 2011; Palfai & Weafer, 2006) and marijuana (Wright & Palfai, 2012) in young university students. There is no lack of studies providing supplementary evidence by confirming the negative association between meaning in life and smartphone addiction in children and adolescents (Qiu et al., 2022) and university students (Çevik et al., 2020). Similarly, it has been concluded that a purpose in life predicts a lower symptomatology of gambling disorders in young university students (M. X. Zhang et al., 2020) and that the link between impulsivity and internet addiction is partially mediated by meaning in life in university students (Y. Zhang et al., 2015).

Bearing in mind the role that meaning and life purpose have in predicting and explaining different behavioral addictions (e.g., mobile phone, gambling, or internet addiction), CB may be considered an attempt at seeking and obtaining meaning. It is likely that a person characterized by emotional instability and life dissatisfaction, as well as by a system of PPs low in meaning, considers buying an objective worth pursuing as it has a restoring effect of their negative mood. However, although, in the short term, buying provides certain enjoyment (Horváth & Adigüzel, 2017), reinforces self-image (Dittmar, 2005) and improves self-esteem (O’Guinn & Faber, 1989), in the long term, it may result in a greater personal dissatisfaction, with a lack of purpose or meaning in life and little sense of direction in life. This existential vacuum linked to a PP system with little meaning probably leads compulsive buyers to exacerbate their buying behavior, thus falling into a vicious circle that perpetuates this behavior. The line of argument is supported by the life stories of persons with buying problems (Eccles, 2002; Otero-López & Villardefrancos, 2009). In their accounts, they often manifest high life dissatisfaction, which they try to manage by owning certain material possessions.

As to the effect of the other personality dimensions on CB, it was found that the meaning of PPs channels the influence of extraversion, agreeableness, openness to experience, and conscientiousness. High scores in these personal traits are associated with high levels of meaning in PPs, which, in turn, are associated with lower involvement in CB behavior. In previous studies (McGregor & Little, 1998), it has been noted that having a system of PPs with meaning has a positive effect on health. Therefore, when discussing our findings, different hypotheses should be put forward—albeit tentatively—on how each trait may impact the meaning given to PPs and their link to CB.

It may be that extraverts, in line with their positive affectivity—cheerfulness, happiness, and enthusiasm—and their optimism (Costa & McCrae, 1992), feel more content with their lives and, therefore, commit to projects they intend to accomplish and enjoy. This pattern is consistent with the results of Little et al. (1992), who noted that extraversion is associated with high meaning in PPs (importance, commitment, self-identity, and enjoyment).

Similarly, kind people tend to embark on projects assessed as important that satisfy their need to establish and maintain their good interpersonal relations conflict-free (Jensen-Campbell & Graziano, 2001). Thus, the meaning of their PPs becomes, again, a protection against CB. Additional support to this approach is provided by the study on values by Uzarska et al. (2021), who concluded that buying addicts place importance on self-enhancement and choose a hedonistic lifestyle at the expense of social relations.

The curious, imaginative, creative, and unconventional mind of people with high openness to experience (McCrae & Costa, 2008) could lead them to embark on projects which they identify with and which reflect their values. The latter point is underpinned by Little et al. (1992), who confirmed in their study that openness to experience establishes a significant, positive link with the dimension of congruency of values of the meaning of PPs. Similarly, this result also agrees with the study by Lavigne et al. (2013), who concluded that people with high levels of openness to experience were more likely to characterize activities involving questioning, learning, and challenging tradition as meaningful.

Determining that the adoption of self-transcendence values by the young is linked to less vulnerability to CB provides additional support to this argument. Specifically, the value of benevolence, which entails a sense of belonging, having meaning in life, and a spiritual life, is associated with a lower incidence of this problem (Tarka & Harnish, 2021).

The fact that the meaning of PPs channels the effect of conscientiousness on CB is easy to understand, taking into account the strong connection of this trait with several dimensions of the meaning of PPs (self-identity, absorption, enjoyment, and importance) (Little et al., 1992). Therefore, it could be argued that a person who is characterized by high dutifulness, a high level of achievement strivings, and a perception of efficacy/feeling of competence values their PPs as highly significant and strives for their objectives, with CB not being one of them.

It also seems reasonable to think that people scoring high on extraversion, agreeableness, openness to experience, and conscientiousness appraise their projects as important and/or significant because these PPs are aimed at attaining inherently rewarding goals; goals which, in turn, entail less risk of CB. More specifically, a pattern observed in previous studies (Otero-López et al., 2021b) confirms that the importance given to inherently rewarding goals of self-acceptance (aimed at satisfying the basic needs of competence and autonomy) and affiliation (aimed at satisfying the need of establishing relations) is a protective factor against CB. In turn, it is people with a high vulnerability to CB who show a lack of confidence in accomplishing the inherently rewarding goals of acceptance, affiliation, and sense of community (Otero-López & Villardefrancos, 2015).

### 4.2. The Mediating Role of the Stress of Personal Projects in the Link Between the Big Five and Compulsive Buying

Another finding of this study is that the appraisal of PPs as stressors becomes a mediating variable of the impact of the traits of neuroticism and conscientiousness on CB. As to the indirect path of influence of neuroticism on CB through the stress of PPs, it should be noted, first, that the effect of this trait on the stress of PPs was significant and positive. This again is consistent with Little et al. (1992), as positive correlations were found between neuroticism and all the dimensions of stress of PPs (difficulty, stress, and challenge). Secondly, the result whereby the appraisal of PPs as stressors increases vulnerability to CB is underpinned by several studies that point at the stress of PPs as a valid predictor of different problems.

For instance, Wallenius (2007) concluded that high personal-project-related stress was linked to lower perceived general health and a greater number of health complaints. On their part, Massey et al. (2009) noted that perceived difficulty in goal attainment and goal frustration were related to low well-being in adolescents, and Nurmi et al. (2009) underscored that a high number of depressive symptoms was associated with a high level of stress of PPs in undergraduates. Particularly relevant is the result obtained by Lecci et al. (2002), who showed that the distress associated with the pursuit of life goals is a significant predictor of heavy alcohol use in university students.

Also of particular interest is a finding from the field of sustainable consumption (Valor & Martínez-de-Ibarreta, 2021) showing that the relationship between PPs and sustainable consumption is mediated by the stress of the sustainable project network. In the field of CB, while it is true that there are no studies that resort to causal approaches where the stress of PPs is the mediating variable, there is extensive empirical evidence that the perception of stress is an important risk factor for CB (Gallagher et al., 2017; Mishra et al., 2023; Thomas et al., 2024; Zheng et al., 2020). Similarly, it should be underscored that the comparison between groups with different levels of CB in the assessment of their PPs (Otero-López et al., 2021c) establishes that the higher the vulnerability to CB, the higher the level of perceived stress of PPs. The tension that the setting of goals generates, the perceived difficulty in achieving these goals, and considering projects to be a challenge are characteristic aspects of the stress of PPs on compulsive buyers.

As to the indirect path of conscientiousness and CB (with the stress of PPs as mediating variable), the following pattern of influences emerges: a low score in conscientiousness involves an appraisal of PPs as stressful, which, in turn, increases the risk of CB. A possible explanation for this result may be that a person with low conscientiousness (poorly organized, not very diligent, and with a scarce capacity for motivation at the start and for the achievement and completion of tasks) (Costa & McCrae, 1992) is likely to show a low level of commitment and accomplishment in their PPs. This lack of efficacy will reinforce thoughts of lack of competence that may contribute to an appraisal of their PPs as stressful and hard to achieve. In the end, this process will result in an undermining of their feeling of personal worth, which will be mitigated through buying behavior. The realization that compulsive buyers are not characterized by using coping strategies aimed at cognitively solving or restructuring situations of stress (Otero-López et al., 2021a; Otero-López & Villardefrancos, 2014) supports this line of argumentation. Supplementary evidence that underscores the importance of some of the facets of this trait (e.g., achievement-striving) as a protection factor against CB is provided by a recent study by Hill et al. (2023), who reported that people who are oriented toward achievement motivation are likely to feel competent in their pursuits, which in turn promotes well-being.

### 4.3. Direct Effects of Neuroticism and Conscientiousness on Compulsive Buying

In this study, neuroticism and conscientiousness, beyond the indirect effect of both traits on CB through the meaning and stress of PPs, were found to have a direct effect on this problem.

Specifically, regarding neuroticism, our results confirm a positive direct effect of this personal dimension on CB. This result was predictable on the basis of what several researchers have reported (Miltenberger et al., 2003; O’Guinn & Faber, 1989; Otero-López & Villardefrancos, 2013a; Yi & Baumgartner, 2023). They link CB to negative emotionality and describe CB behavior as an attempt at repairing or compensating for prior negative emotional states. Moreover, prior research provides clear supportive evidence of the positive link between certain facets which characterize neuroticism, such as anxiety (Weinstein et al., 2015), depression (Kyrios et al., 2013), impulsivity (Mishra et al., 2023), or vulnerability to stress (Zheng et al., 2020) and this behavioral problem. In addition, our finding with regard to neuroticism is clearly in keeping with some studies that put forward a variety of causal proposals (Mowen & Spears, 1999; Otero-López & Villardefrancos, 2013a; Shehzadi et al., 2016) and that confirm the importance of this personal dimension in explaining CB by corroborating its direct positive impact on this problem.

Conscientiousness is consolidated, as shown by our results, as a dimension of personality that exerts a direct negative impact on CB. This finding is in line with a number of studies (Achtziger et al., 2015; Jiang & Shi, 2016) that show that a deficit in self-control, with what it entails (difficulty to plan, organize, and perform tasks), is a significant predictor of CB. It is also in line with a recent study (Yi & Baumgartner, 2023) that shows that the lack of the trait of self-control primarily promotes the dimension of excessive buying in CB. There is no shortage of studies, either, that confirm the explanatory power of conscientiousness for CB from the perspective of causal approaches. Thus, from the pioneering study by Mowen and Spears (1999) with university students to later studies conducted on general populations from different geographical domains (Otero-López & Villardefrancos, 2013a; Shehzadi et al., 2016; Tarka et al., 2022), conscientiousness becomes a personality factor that has a direct negative impact on engaging in CB.

One final point, revisiting the findings obtained on the direct effect of neuroticism and conscientiousness, has to do with the possibility of considering CB, like other authors have done (Tarka et al., 2022), to be a maladaptive expression of personality, because of both the marked influence of neuroticism and the deficit of conscientiousness. Another suggestion has to do with the high level of consensus among experts with regard to these two of the diagnostic criteria of CB (Müller et al., 2021), which seems to underscore the relevance of these two personality traits for this problem. Thus, the criterion of using buying/shopping to regulate internal states (e.g., negative moods such as nervousness, tension, negative feelings) would support the importance of neuroticism, while the criterion of diminished control over buying/shopping would be in line with low scores in conscientiousness.

## 5. Strengths and Limitations

This study has major strengths that contribute to gaining a deeper understanding of the nature of CB. It is the first time in this field that an empirical test of a model of relationships analyzes whether PPs (particularly the dimensions of meaning and stress) mediate the relation between the Big Five and CB. Or, to put it differently, considering, on the basis of models and/or theoretical schemes of great relevance in the study of personality (McAdams, 2013; McCrae & Costa, 2008), the basic dispositions of personality (Big Five) jointly with PPs (action units which imply “doing something”) to explain CB is a pioneering avenue in research in the field of CB. Selecting a sample of university students is also a strength of this study. It should be borne in mind that the university stage entails major changes in the PPs of the young. Consequently, examining how the meaning and stress of PPs impact CB and, in turn, channel the impact of the Big Five in this age bracket seems to be a necessary step if we want to understand the dynamics associated with the onset and development of this behavioral problem.

However, this research has some limitations, which may be addressed in future research. First, the cross-sectional nature of this study limits the possibility of establishing causal relations. Although the order of influences that we have established (Big Five–PP-CB) is solidly supported by other proposals (Little, 2000; McAdams, 2013; McCrae & Costa, 2008), only the development of prospective and longitudinal studies will permit the establishment of valid conclusions regarding the causal relationships between the variables we have analyzed. Second, all the variables were measured by self-reports. Supplementary methods of data collection (e.g., clinical interview, tracking on actual behavior, reports by third parties) would be desirable. Moreover, beyond the assessment of personality traits and characteristic adaptations, it would be advisable to obtain information from autobiographical narratives to have a more complete view of the compulsive buyer. Finally, the selection of university students from a specific sociodemographic context limits generalization to other age brackets and cultures. Considering that PPs are extended sets of personality salient actions in context (Little, 2015), it would be particularly desirable to replicate the model of relationships used in other samples and cultural contexts (without losing sight of the evaluation of the role of other variables, such as gender, age, social class, and educational level) to gain a more comprehensive understanding of CB.

In sum, and despite the limitations mentioned, the results of this study support the suitability of integrating different types of personal units—dispositional traits and purpose-oriented units—in explaining CB. In addition, these findings suggest that attention should be paid to the dispositional equipment of compulsive buyers (particularly in the case of high levels of neuroticism and low conscientiousness) and their PPs (enhancing their meaning and reducing the stress they generate would be a healthy practice). This should be accomplished by approaching this problem from both a preventive and interventional approach.

## 6. Conclusions, Practical Implications, and Future Perspectives

The aim of this study, which originated in the recognition of the need to adopt an integrative perspective in the study of personal determinants that explain CB, was to empirically test a causal model of relationships where the mediating role of PPs (specifically, the dimensions of meaning and stress) is examined in link between the Big Five personality traits and CB. Our findings provide evidence of the mediating role of the meaning and stress of PPs in the influence of the Big Five personality traits on CB. Specifically, our results confirm that while meaning screens the influence of each of the Big Five factors of personality, stress channels the influence of neuroticism and conscientiousness. Besides these indirect paths (through the meaning and stress of projects), neuroticism and conscientiousness also have a direct effect on CB.

Some implications might be derived from the results of this research. Generally speaking, our findings are potentially useful not only to further knowledge of the dynamics of influence between the Big Five personality traits and CB but also to suggest actions that may be included in prevention and/or treatment programs addressing this problem.

Indeed, the confirmation that the meaning of PPs channels the influence of the Big Five in CB suggests the need to underscore and act on the perceived meaning of the system of PPs of those most vulnerable to CB. Encouraging young people from socio-educational domains to involve themselves in projects they consider important, self-defining, and consistent with their values is one preventive suggestion to enhance the meaning of their projects, thus reducing the probability of engaging in problem behaviors (CB, in this case). Including actions aimed at promoting reflection on the part of compulsive buyers in treatment programs with regard to the importance compulsive buyers attribute to extrinsic personal goals (e.g., image, popularity, conformity) to the detriment of intrinsic goals (self-acceptance, affiliation, and community feeling) and reflect on to what extent this appraisal impacts their buying behavior is another option that should be taken into account. There are, in this vein, a number of programs aimed at achieving personal goals (Sheldon et al., 2002), to explore, discover, and enact a sense of purpose (Dik et al., 2011) and, most particularly, to encourage intrinsic goals to reduce a materialistic goal orientation (Parker et al., 2020), which have become key milestones in the intervention domain.

Other potential lines of action may be put forward on the basis of the indirect effect, through the stress of PPs, of neuroticism and conscientiousness on CB. Assuming that one of the functions of affect is to direct and energize goal-directed behavior, it would be desirable to reduce the negative emotionality that neuroticism entails by resorting to a variety of strategies (e.g., relaxation, visualization, mindfulness) to reduce the stress associated with the PPs of the young, and, in sum, reduce their engagement in CB. Another healthy path would be to foster positive emotions, as positive affect broadens an individual’s thought–action repertoires (Fredrickson, 2001) and promotes involvement in self-defining, stress-free projects. The reduction in CB levels obtained by Lekavičienė et al. (2022), when they applied a training program in emotional intelligence, is a valuable example in this regard. As to conscientiousness, several of its facets (e.g., need for achievement, deliberation, and self-discipline) should be promoted. This would reduce the probability of engaging and/or progressing in CB, as personal satisfaction increases as a result of accomplishing PPs and the appraisal of stress associated with their accomplishment is reduced. In addition, it is to be expected that training in the typical sequence of stages through which projects progress en route to completion—inception, planning, action, and termination (Little, 1983)—proves useful to strengthening the mechanisms of self-regulation of behavior and reducing the appraisal of PP-related stress.

In sum, the results of this study have led to the identification of some aspects in relation to both the Big Five and PPs which may operate as possible targets in the design of treatment programs: (a) promoting techniques for the adequate management of negative emotions (e.g., anxiety, depression, hostility) associated with high levels of neuroticism and frequently linked to CB; (b) developing specific skills to identify PPs which generate high levels of stress and teach–train in proactive coping styles (e.g., problem solving, cognitive restructuring) to reduce such experiences; (c) increasing conscientiousness by promoting some of its facets (deliberation, order, sense of competence, discipline) to encourage the appraisal of PPs as valuable (with meaning) and rather stress-free so that, ultimately, there is greater control over behavior (e.g., compulsive buying); and (d) encouraging, in people with buying problems, the ability to engage in reflection regarding their system of goals by making them aware that the projects appraised as being of little usefulness and greatly stressful often channel emotional distress and increase the probability of CB.

## Figures and Tables

**Table 1:** Regression analyses performed to test for mediator effects of the meaning and stress of PPs.

	Big Five as Predictors of Meaning in PPs *Beta*	Big Five as Predictors of Stress in PPs *Beta*	Big Five as Predictors of CB *Beta*	Big Five and Meaning and Stress of PPs as Predictors of CB *Beta*
Neuroticism	−0.13 **	0.21 ***	0.35 ***	0.26 ***
Extraversion	0.11 **	0.02	0.04	0.05
Openness	0.13 **	−0.09 *	−0.02	0.03
Agreeableness	0.15 **	−0.08	−0.06	−0.01
Conscientiousness	0.26 ***	−0.21 ***	0.29 ***	−0.18 ***
Meaning of PPs				−0.22 ***
Stress of PPs				0.27 ***
Explained variance	17.4	12.3	26.1	35.2

* *p* < 0.05; ** *p* < 0.01; *** *p* < 0.001.

**Table 2:** Correlations, means, and standard deviations of all the variables studied.

	1	2	3	4	5	6	7	8
1. Compulsive buying	1							
2. Meaning PP	−0.34 ***	1						
3. Stress PP	0.40 ***	−0.09 *	1					
4. Neuroticism	0.42 ***	−0.20 ***	0.26 ***	1				
5. Extraversion	−0.01	0.16 ***	−0.02	−0.07	1			
6. Openness	−0.08	0.17 ***	−0.09 *	0.02	0.30 ***	1		
7. Agreeableness	−0.16 ***	0.22 ***	−0.15 ***	−0.10 *	−0.01	0.06	1	
8. Conscientiousness	−0.38 ***	0.32 ***	−0.27 ***	−0.21 ***	0.03	0.02	0.21 ***	1
								
*Mean*	29.96	7.35	5.49	59.38	48.09	52.26	39.95	40.99
*SD*	7.34	0.77	1.22	10.53	10.73	10.01	8.95	9.33

* *p* < 0.05; *** *p* < 0.001.

## Data Availability

The data that support the findings of this study are available on request from the corresponding author. The data are not publicly available due to privacy restrictions.
